# Periostracum Cicadae Extract and N-Acetyldopamine Regulate the Sleep-Related Neurotransmitters in PCPA-Induced Insomnia Rats

**DOI:** 10.3390/molecules29153638

**Published:** 2024-07-31

**Authors:** Dongge Wang, Tingjuan Wu, Jinghui Jin, Yanpo Si, Yushi Wang, Xiaojia Ding, Tao Guo, Wenjun Wei

**Affiliations:** 1 School of Pharmacy, Henan University of Chinese Medicine, Zhengzhou 450046, China; donggewang01@163.com (D.W.); wutj2011@163.com (T.W.); jjh5616@163.com (J.J.); 13937187124@163.com (Y.S.); 2 Henan Engineering Research Center of Medicinal and Edible Chinese Medicine Technology, Zhengzhou 450046, China; 3 Bencao Academy, Henan University of Chinese Medicine, Zhengzhou 450046, China; wys051121@163.com (Y.W.); 18837296832@163.com (X.D.)

**Keywords:** insomnia, periostracum cicadae, N-acetyldopamine

## Abstract

Insomnia is the second most prevalent mental illness worldwide. Periostracum cicadae (PC), as an animal traditional Chinese medicine with rich pharmacological effects, has been documented as a treatment for children’s night cries, and later extended to treat insomnia. This study aimed to investigate the effects of PC extract and N-acetyldopamine compounds in ameliorating insomnia. The UPLC-ESI-QTOF-MS analysis determined that PC extract mainly contained N-acetyldopamine components. Previously, we also isolated some acetyldopamine polymers from PC extract, among which acetyldopamine dimer A (NADA) was present in high content. Molecular docking and molecular dynamic simulations demonstrated that NADA could form stable complexes with 5-HT1A, BDNF, and D2R proteins, respectively. The effects of PC extract and NADA on insomnia were evaluated in the PCPA-induced insomnia model. The results indicated that PC extract and NADA could effectively ameliorate hypothalamic pathology of insomnia rats, increase the levels of 5-HT, GABA, and BDNF, and decrease the levels of DA, DOPAC, and HVA. Meanwhile, the PC extract and NADA also could significantly affect the expression of 5-HT1A, BDNF, and DARPP-32 proteins. This study proved that PC extract and acetyldopamine dimer A could effectively improve PCPA-induced insomnia in rats. It is speculated that the main pharmacological substances of PC were acetyldopamine components.

## 1. Introduction

The World Health Organization statistics show that the global incidence of insomnia accounts for about 35%, which has become the second most common mental illness. In China, the proportion of insomnia patients is as high as 38%, of which approximately 300 million middle-aged people suffer from sleep disorders, and the trend is gradually increasing [1,2]. Insomnia is always associated with neurodegenerative diseases, cardiovascular issues, type II diabetes mellitus, anxiety, depression, substance abuse, and suicidal ideation [1,2,3]. Currently, benzodiazepines and melatonin receptor agonists, as well as anti-convulsants, anti-depressants, and anti-psychotics with a hypnotic effect, are used in clinics to treat insomnia [4]. However, some of the above drugs have many adverse effects, such as tolerance, addiction, withdrawal syndrome, daytime residual effects, and rebound after discontinuation [2,4]. Therefore, there is an urgent need to develop new drugs with higher efficacy and fewer side effects for the targeted treatment of insomnia.

Traditional Chinese medicines are gradually accepted by the world for their safety and efficacy. Many plant-based drugs have been used in the treatment of insomnia [5]. The use of insects as folk medicine has a long history in some countries. However, medicinal insects have been largely ignored compared to plant-based drugs. Although medicinal insects play an important role in the treatment of refractory diseases due to their unique and rich pharmacological activities, there is little research on their therapeutic effects, especially on small molecule compounds from medicinal insects [6,7,8,9]. Therefore, medicinal insects and insect-derived substances have great potential for exploring new drug sources and effectively expanding the screening scope of natural drugs.

Periostracum cicadae (PC), the cast-off shell of the *Cryptotympana pustulata* Fabricius belonging to the cicadidae, is an animal-based traditional Chinese medicine in Korea and China [10]. PC possess many interesting pharmacological and physiological activities, such as diaphoretic, anti-convulsive, sedative, anti-pyretic, and anti-allergic effects [11,12]. PC is originally described in Ming-I-Pieh-Lu (an ancient Chinese medical book dating to the Han Dynasty), which is used to treat convulsions, the nocturnal crying of children, delirium, and feverish chills [13]. Because of its efficacy in treating children’s convulsions and nocturnal crying, PC was later extended to treat insomnia, which further expands the scope of the clinical applications of PC. Simultaneously, clinical applications also have proven that PC has a good sedative effect [14,15,16,17]. Modern pharmacological studies have determined that PC extract possesses antioxidant, anti-inflammatory, anti-anaphylactic, anti-convulsive, and sedative–hypnotic activities [13,18,19]. PC can treat insomnia in clinical practice [20,21], but there are few reports on the pharmacological material basis and mechanism of action in ameliorating insomnia.

Ultra-performance liquid chromatography electrospray quadrupole time-of-flight mass spectrometry plays a significant role in identifying and analyzing components of traditional Chinese medicine extracts [22]. Previous investigations have shown that PC mainly contains N-acetyldopamine components [12,23]. In this study, UPLC-ESI-QTOF-MS was used to analyze the main components of the 70% methanol extract of PC, which provided a theoretical basis for clarifying the pharmacological substance basis of PC in improving insomnia. Meanwhile, we found that N-acetyldopamine components of this PC extract were mainly enriched in the 80% methanol fraction (PC-80) after being treated with macroporous resin [24]. A lot of N-acetyldopamine compounds were also obtained from this fraction, among which the content of N-acetyldopamine dimer A (NADA) is relatively high.

Therefore, in order to explore the effect of PC extract and NADA on ameliorating insomnia, as well as the material basis and mechanism of action for improving insomnia, p-chlorophenylalanine (PCPA)-induced sleep deprivation in SD rats was established. The animal experiment results showed that PC extract and NADA can improve insomnia in rats, possibly by affecting the neurotransmitters related to insomnia. It is speculated that the main pharmacological substances of PC for improving insomnia were acetyldopamine components.

## 2. Results and Discussion

### 2.1. UPLC-ESI-QTOF-MS Analysis of PC Extract

**Figure 1:**
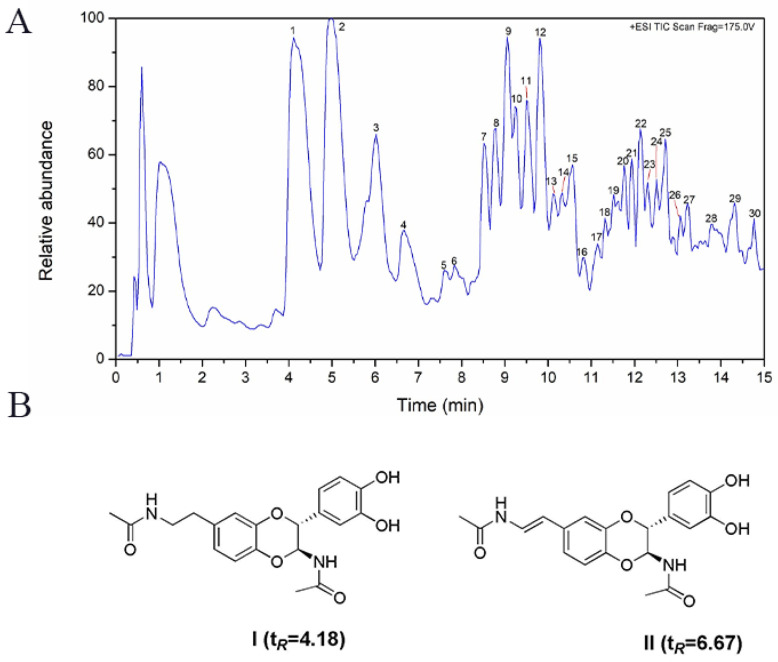
(**A**) Total ion chromatogram (+ESI) of PC extract; (**B**) The structures of standard Ⅰ and standard Ⅱ.

In this study, UPLC-ESI-QTOF-MS was used to analyze the main chemical components in PC extract. The analysis result showed that the 70% methanol extract of PC mainly contained *N*-acetyldopamine components, especially polymers, including dimers, trimers, tetramers, and pentamers, as shown in Figure 1 and Table 1. In addition, these polymers were either isomers or epimers of each other and their separation and identification were difficult. Our previous research also found that acetyldopamine analogues were enriched in the 80% methanol fraction (PC-80) after being treated with macroporous resin. A lot of acetyldopamine dimers and trimers were isolated from this PC-80 fraction, of which NADA possessed a high content. Therefore, the PC extract, PC-80 fraction, and monomer compound (NADA) were all investigated in animal experiment.

### 2.2. Effect of PC on Body Weight and Behavior of Rats

**Figure 2:**
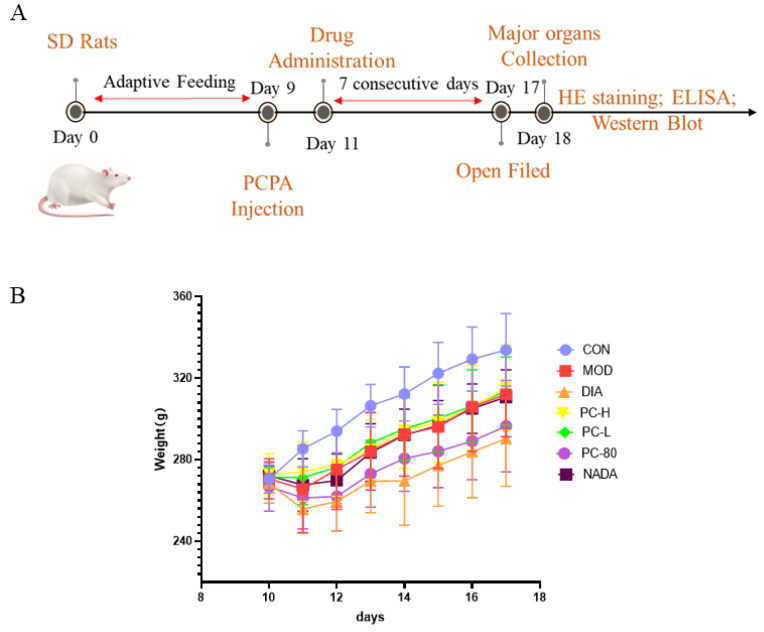
(**A**) Schematic representation of the animal experiment. (**B**) The effects of crude extract and monomer compound from PC on weight changes in PCPA-induced insomnia rats (*n* = 10 per group). Abbreviations: CON: control group; MOD: model group; DIA: diazepam; PC-H: high-dose PC extract group; PC-L: low-dose PC extract group; PC-80: PC-80 fraction group; NADA: N-acetyldopamine dimer A group.

**Figure 3:**
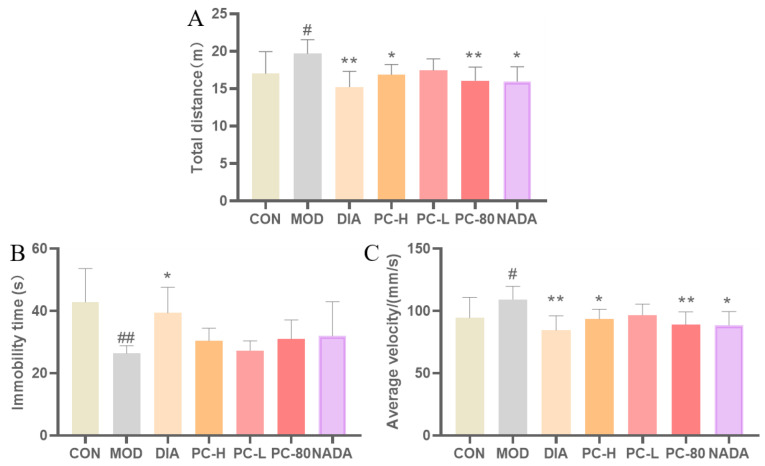
The open-field test of PCPA-induced insomnia rats. (**A**) Total distance of the rats. (**B**) Immobility time of the rats. (**C**) Average velocity of the rats. (Values are presented as the means ±deviation for each group, *n* ≥ 6 per group. Compared with the control group, #: *p* < 0.05, ##: *p* < 0.01; compared with the model group, *: *p* < 0.05, **: *p* < 0.01.). Abbreviations: CON: control group; MOD: model group; DIA: diazepam; PC-H: high-dose PC extract group; PC-L: low-dose PC extract group; PC-80: PC-80 fraction group; NADA: N-acetyldopamine dimer A group.

5-HT is essential for regulating the neurotransmitter system in the brain, and its shortage may lead to mental disorders. PCPA is a tryptophan hydroxylase (TPH) inhibitor that can selectively inhibit TPH, thus blocking the synthesis of 5-HT. PCPA is introduced via intraperitoneal injection to establish a sleep deprivation animal model, which is the most classic insomnia model [25,26]. In this study, the PCPA-induced insomnia model was applied to study the pharmacological action mechanism of PC crude extract. As shown in Figure 2, the rats in all groups except the control group lost weight significantly after the injection of PCPA. From the third day, the weight of each administration group began to gradually increase. The organ index of the rats is the ratio of organ weight to body weight. The changes in organ index often reflect the comprehensive toxicity of drugs to an organ, which can be evidence of the possibility of histopathological changes [27]. In addition, compared with the blank group, the brain and kidney organ indices of the PCPA model rats increased in all groups (Table 2), which indicated that the modelling did not cause organic damage to the rats. The organ indices of the insomnia rats in the other dosing groups showed a trend of recovery, which demonstrated the safety of the PC extracts. Then, the open-field test was used to explore the behaviors of the rats, as presented in Figure 3. The results demonstrated that the rats in model group showed abnormal excitement, which was also a typical feature of the PCPA-induced insomnia model in rats. Moreover, the total moving distance and average velocity of the model group rats significantly increased compared with the blank group (*p* < 0.05), and the immobility time was significantly reduced in the model group (*p* < 0.01). The above results indicated that the PCPA-induced insomnia model had been successfully established. Compared with the model group, the trajectories, total distance, and average velocity of the rats in each administered group were significantly reduced and the immobility time of the rats in each treatment group had increased. Thus, PC extract and *N*-acetyldopamine dimer A could ameliorate abnormal behaviors in insomnia rats.

### 2.3. Effect of PC on the Hypothalamus Neuronal Cells of Rats

Sleep is regulated by the circadian rhythm and homeostatic mechanisms, which are dominated by the central nervous system (CNS) [28]. The hypothalamic–pituitary–adrenal (HPA) axis plays important roles in modulating sleep. The HPA axis, starting from the hypothalamus and stimulating the production of glucocorticoids, is the main stress axis of the body [29]. Sleep has a close and reciprocal association with the ability of the HPA axis to operate [30]. In general, HPA activation causes lighter sleep and increases nocturnal awakening, while insufficient sleep has been shown to increase the basal activity of the HPA axis [31]. PCPA is a 5-HT synthesis inhibitor that blocks the synthesis of 5-HT, leading to the loss of the circadian rhythm of sleep. This process is usually accompanied by dysfunction of the HPA axis [32]. Thus, hypothalamic tissues of the PCPA-induced insomnia rats were chosen for further experimental research in this paper.

**Figure 4:**
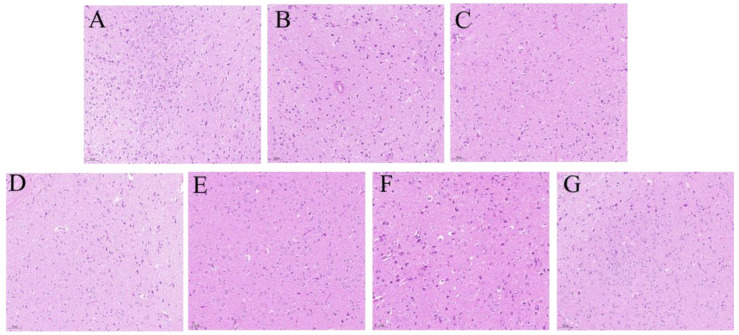
Pathological section of the hypothalamus stained by HE (*n* = 3) (magnification: ×20). (**A**) CON, (**B**) MOD, (**C**) DIA, (**D**) PC-H, (**E**) PC-L, (**F**) PC-80, and (**G**) NADA. Abbreviations: CON: control group; MOD: model group; DIA: diazepam; PC-H: high-dose PC extract group; PC-L: low-dose PC extract group; PC-80: PC-80 fraction group; NADA: N-acetyldopamine dimer A group.

The histopathological examination of the hypothalamus showed that the hypothalamic neuronal cells were regularly arranged, intact, and clearly visible in the control group (Figure 4A). However, the neuronal cells in the model group were loosely arranged as shown in Figure 4B, and these cells were also tapered or polygonal, suggesting pathological changes in hypothalamic cells after injecting PCPA. Compared with the model group, the cell state of the treatment groups was improved and the number of deformed cells decreased, as shown in Figure 4C–G. In particular, the improvement status of the PC-80 and NADA groups was more significant than the PC-L and PC-H groups. The above results indicated that PC extract and NADA could repair injured neuronal cells in the hypothalamus of rats.

### 2.4. Effect of PC on 5-HT, DA, GABA, HVA, DOPAC, BDNF Levels in Hypothalamus

Sleep–wake is a complex physiological process that is regulated by the activity of multiple parts of the brain, among which the neurotransmitter system plays an important role [33]. The disturbances in neurotransmitters in the brain are widely accepted to be associated with insomnia [26]. Many endogenous neurotransmitters are involved in sleep mechanisms in the brain, including monoamines and noradrenergic and cholinergic neurotransmitters [34]. Dopamine (DA), gamma-aminobutyric acid (GABA), and serotonin (5-HT) play important roles in maintaining wakefulness and sleep. GABA and 5-HT are two of the most important sleep promoting neurotransmitters in the brain, which can reduce the activity of neurons and regulate the function of nerve cells. However, DA usually conducts excitement and nerve impulses, which is related to wakefulness [35,36]. BDNF is a major factor that regulates the process of synaptogenesis and plasticity, which is widely found in the CNS. BDNF can regulate the plasticity of synaptic nerves in the body, and promote axonal growth as well as neuronal repair. Simultaneously, BDNF is involved in regulating sleep–wake homeostasis [37].

**Figure 5:**
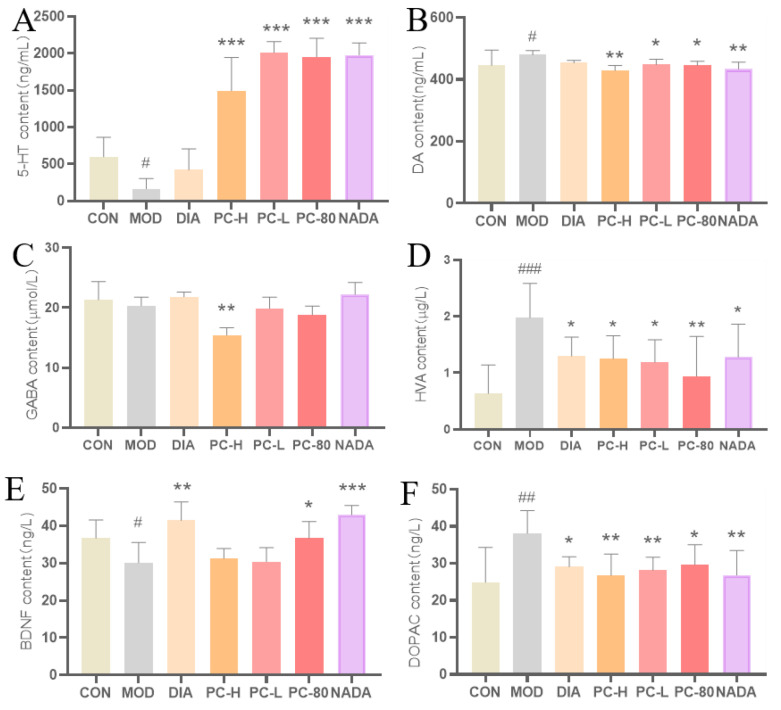
The effects of PC on neurotransmitter and metabolite levels in rat hypothalamus. (**A**) 5-HT, (**B**) DA, (**C**) GABA, (**D**) HVA, (**E**) BDNF, and (**F**) DOPAC. Values are presented as the means ± deviation for each group, *n* ≥ 6 per group. Compared with the control group, #: *p* < 0.05, ##: *p* < 0.01, ###: *p* < 0.001; compared with the model group, *: *p* < 0.05, **: *p* < 0.01, ***: *p* < 0.001. Abbreviations: CON: control group; MOD: model group; DIA: diazepam (Positive control group); PC-H: high-dose PC extract group; PC-L: low-dose PC extract group; PC-80: PC-80 fraction group; NADA: N-acetyldopamine dimer A group.

In this study, the levels of the neurotransmitters 5-HT, DA, GABA, and BDNF, as well as the metabolites HVA and DOPAC of DA, were detected in the hypothalamus of rats. Compared with the control group, the content of 5-HT and BDNF in the model group rats decreased significantly (*p* < 0.05), and the content of DA, HVA, and DOPAC increased significantly (Figure 5). The content of GABA also declined, but there was no significant difference, as presented in Figure 5C. The significant differences between the model and control groups indicated that the synthesis of 5-HT was specifically blocked, and the disorder of the neurotransmitter system was caused after injecting PCPA for insomnia modeling. Compared with the model group, 5-HT and BDNF levels were raised in the DIA, PC-H, PC-L, PC-80, and NADA groups, while the levels of DA, HVA, and DOPAC were significantly reduced. Furthermore, the regulatory effects in the PC extract administration groups were equivalent to that of the diazepam group in DA, HVA, and DOPAC levels. It can be inferred that there may be similarities in the effect of PC extract and diazepam in the treatment of insomnia. Both them may treat insomnia by regulating the inhibitory neurotransmitters and excitatory neurotransmitters. In addition, the effect was more pronounced in the low-dose group than the high-dose group. It was possible that the administered dose in the high-dose group was higher than the effective dose. Additionally, the PC-80 and NADA groups had better effects than other groups. Previously, it had been proven that the PC-80 fraction was mostly dominated by acetyldopamine analogues [24], which may be the effective substances for PC to ameliorate insomnia. Indeed, N-acetyldopamine dimer A (NADA group) can also significantly affect the levels of 5-HT, DA, GABA, BDNF, HVA, and DOPAC. However, there was no significant difference in GABA content among the treatment groups (Figure 5C). These results suggested that PC extract and the N-acetyldopamine component have effects on the neurotransmitter system, and the sleep-promoting effect may be mainly achieved by regulating the contents of the 5-HT and DA neurotransmitters.

### 2.5. Molecular Docking Analysis

In the CNS, 5-HT1_A_ receptors among 5-HT receptor subtypes are mainly involved in sleep regulation. The 5-HT1_A_ receptor is essential for regulating the neurotransmitter system in the brain. Its deletion or over-expression may lead to mental disorders [38,39]. GABA is the main inhibitory neurotransmitter in the CNS and its levels are correlated with the occurrence of insomnia. GABA may inhibit arousal systems to promote sleep by binding to the GABA_A_ receptor [40]. There are generally three types of GABA receptors, including GABA_A_, GABA_B_, and GABA_C_, with GABA_A_ being the main receptor type involved in sleep in the brain [41,42]. The physiological actions of GABA appear mostly through the GABA_A_ receptor, which plays an important role in sedation, sleep, and anesthesia [43,44]. It is well-known that activation of the GABA_A_ receptor is beneficial for sleep [45]. Extensive connections between DAergic neurons and sleep–wake brain regions suggest that the DA system may modulate sleep–wake. Dopamine receptors are divided into D1 and D2 class receptors, with D2 class receptors having a much greater affinity for endogenous DA than D1 class receptors. D2R plays an essential role in the maintenance of wakefulness. It has been shown that knockdown of D2R in animals as a whole lead to a significant reduction in arousal, accompanied by non-rapid eye movement and rapid eye movement [46]. It is evident that D2R plays a key role in maintaining arousal. BDNF is a brain-derived neurotrophic factor, which plays an important role in sleep regulation. Prolonged sleep deprivation causes a decrease in BDNF levels and increasing BDNF levels during wakefulness promotes sleep activity in the slow-wave sleep phase [37].

**Figure 6:**
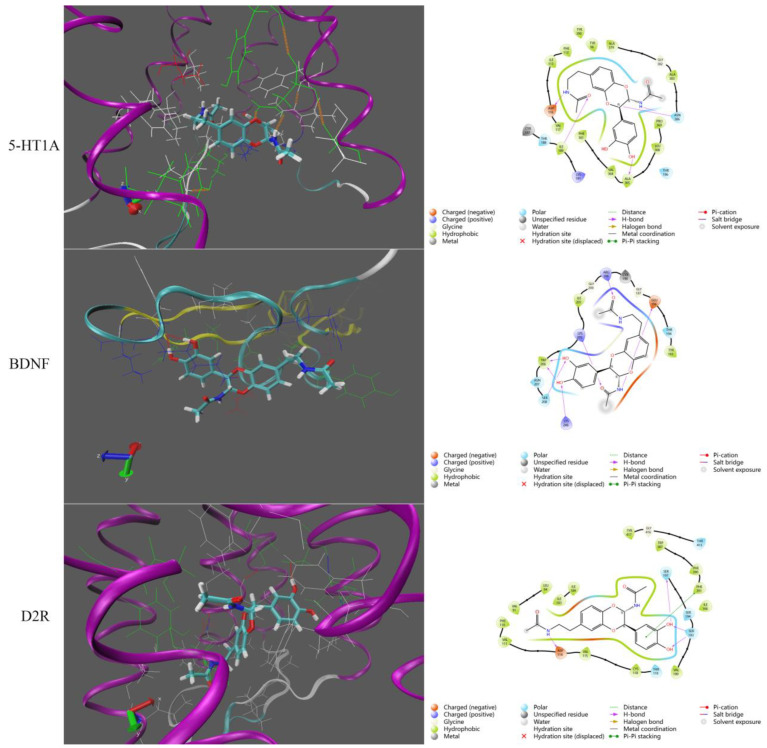
The molecular docking results between small molecule NADA and 5-HT1_A_, BDNF and D2R proteins, respectively.

**Figure 7:**
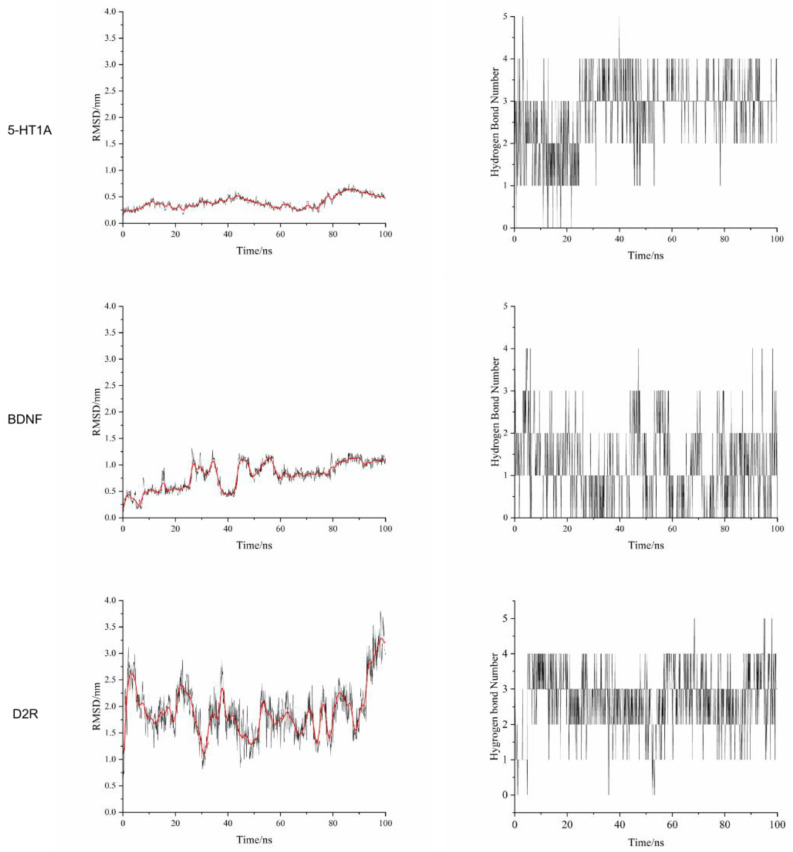
Molecular dynamic simulation results between small molecule NADA and 5-HT1_A_, BDNF and D2R proteins, respectively.

In order to explore the possible protein targets of PC extract and NADA, the monomer compound NADA was used to perform molecular docking with 5-HT1_A_, BDNF, D2R, and GABA_A_ proteins, respectively. The molecular docking result demonstrated that small molecular NADA can combine well with protein receptors D2R, BDNF, and 5-HT1_A_ to form stable ligand–protein complexes, but it cannot form stable complexes with GABA_A_. The two-dimensional pattern displayed that NADA mainly binds to multiple amino acid sites of D2R, BDNF, and 5-HT1_A_ receptor proteins through hydrogen bonds (Figure 6). To further assess the stability of the binding conformation of NAND to the target protein, a molecular dynamic simulation was used to evaluate the binding stability of the ligand–protein complex. RMSD represents the change of distance and time between small molecules and ligands. The hydrogen bond number refers to the variation in the number of hydrogen bonds between small molecules and proteins during the molecular dynamic simulation process. As shown in Figure 7, the RMSD values of three docking results did not show a significant increasing trend over time. Especially for the simulation between protein 5-HT1_A_ and NADA, the range of RMSD value changes is smaller, indicating a more stable combination of this protein and small molecule. The numbers of hydrogen bonds of these ligand–protein complexes were greater than 0. Therefore, the molecular dynamic simulation results further demonstrated that the small molecule NADA can stably bind to the active pockets of the D2R, BDNF, and 5-HT1_A_ proteins.

### 2.6. Effect of PC on the Expression of 5-HT1_A_, BDNF and DARPP-32 Protein in the Hypothalamus of Rats

To evaluate the modulation effect of the PC extract on 5-HT1_A_, D2R, GABA_A_, and BDNF, the expression of these receptors in the hypothalamus of the rats was determined by Western blotting. The results indicated that the PC extract and NADA could significantly affect the content of 5-HT1_A_ and BDNF proteins. However, the effects of PC extract and NADA on the GABA_A_ protein had no significant difference, and this experimental result was mutually verified with the molecular docking results. Unfortunately, the protein content of D2R was very low, thus the DARPP-32 protein expression was further tested. DARPP-32 is a dopamine and cAMP-regulated phosphoprotein. The expression of DARPP-32 plays a pivotal role in dopamine neurotransmission, which is expressed in dopaminoceptive neurons [47].

**Figure 8:**
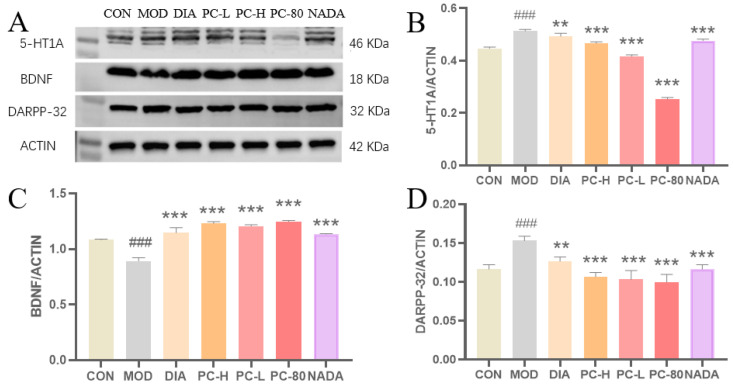
The effects of PC on protein expression in the hypothalamus of rats. (**A**) Protein level normalized with ACTIN. (**B**) Comparison of 5-HT1_A_ receptor protein expression in each group. (**C**) Comparison of BDNF receptor protein expression in each group. (**D**) Comparison of DARPP-32 protein expression in each group. (Values are presented as the means ± standard deviation for each group, *n* = 3. Compared with the control group, ###: *p* < 0.001; compared with the model group, **: *p* < 0.01, ***: *p* < 0.001.). Abbreviations: CON: control group; MOD: model group; DIA: diazepam; PC-H: high-dose PC extract group; PC-L: low-dose PC extract group; PC-80: PC-80 fraction group; NADA: N-acetyldopamine dimer A group.

In this study, the 5-HT level significantly reduced in rats after the injection of PCPA (Figure 5A), and its corresponding receptor 5-HT1_A_ showed over-expression (Figure 8B). Simultaneously, DARPP-32 protein expression was elevated (*p* < 0.001) and BDNF expression decreased in the model group (*p* < 0.001). As presented in Figure 8, DARPP-32 and 5-HT1_A_ protein expressions were reduced and BDNF expression was up-regulated in DIA, PC-H, PC-L, PC-80, and NADA groups, in contrast to the model group. In addition, the protein expression of each group in the treatment group was similar to that of the DIA group. These results provide evidence that PC extract and NADA can affect the expression levels of these sleep-related proteins.

## 3. Materials and Methods

### 3.1. Materials

Periostracum cicadae (PC) was purchased from Zhengzhou Herbal Market, Zhengzhou City, Henan Province, China, in October 2022. This medicinal herb was authenticated by Professor Guo Tao from the Henan University of Chinese Medicine and has been deposited in the Henan Engineering Research Center of Medicinal and Edible Chinese Medicine Technology (voucher specimen: PC20221001). PC was extracted twice with 70% ethanol reflux for 2 h each time. The extracted solution was concentrated under reduced pressure to obtain the crude extract of PC. The extraction rate of the PC crude extract was 5.71%. The crude extract was segmented on HP-20 macroporous resin, eluting with a 30%, 50%, 80%, and 100% methanol solution, respectively. An 80% methanol eluting portion was evaporated to produce the PC 80% crude extract fraction (PC-80), with the extraction rate being 1.43%.

### 3.2. Reagents

PCPA was purchased from Sigma-Aldrich. Diazepam was used as the positive control drug and manufactured by Huazhong Pharmaceutical Co., Ltd. (Xiangyang, Hubei, China) with lot No. H42021528. Chloral hydrate for rat anaesthesia was purchased from the Shanghai Maclin Biochemical Technology Co., Ltd. (Shanghai, China) with lot no. C14975135. The assay kit for the determination of DA was bought from Nanjing Jiancheng Biotechnology Co., Ltd. (Nanjing, Jiangsu, China). The assay kit for the determination of 5-HT was purchased from Elabscience (Wuhan, Hubei, China). GABA, BDNF, HVA, DOPAC assay kits were bought from Enzyme-free (Yancheng, Jiangsu, China). The RIPA lysis buffer, HE staining kits, BCA protein quantitative detection kit, and SDS-PAGE gel preparation kit were purchased from Servicebio Technology Co., Ltd. (Wuhan, Hubei, China). All other chemicals and reagents were of analytical purity.

### 3.3. UPLC-ESI-QTOF-MS Conditions

The Agilent 1290 UPLC system coupled to Agilent 6550 iFunnel Q-TOF mass spectrometer (Agilent Technologies Inc., Santa Clara, CA, USA) was selected to analyze the samples. The LC system consists of Agilent 1290 Infinity G4220A binary pumps, Agilent 1290 Infinity G1316C column oven, Agilent 1290 Infinity G4226A sampler and Agilent 1290 Infinity G1330B autosampler column chamber.

A Waters BEH C18 column (2.1 mm × 100 mm, 1.7 μm, Waters Corporation, Milford, MA, USA) was used with a mobile phase consisting of eluent A (H_2_O solution contained 0.1% HCOOH) and eluent B (acetonitrile solution). The mobile phase was used with a gradient elution condition of 0.01–1 min, 90% A; 1–60 min, 100% B. The flow rate was 0.3 mL/min and the volume of the injected sample was 5 μL. The mass spectrometric detector was operated in the positive ESI mode and negative ESI mode. The mass range was set at *m*/*z* 100–1500. All data acquisition and analysis were controlled by the Agilent MassHunter Qualitative Analysis software (version: B7024.0).

### 3.4. Animal Administration

SPF-grade SD male rats (180–220 g) from Beijing Vitonglihua Experimental Animal Technology Co., Ltd. were used for the PCPA-induced insomnia model. The laboratory animal license number is SCXK (Hubei) 2022-0030 and animal quality certificate number is NO. 422023600002711. The animal protocol was approved by the Scientific Ethics Committee of the Center for Laboratory Animals in Henan University of Chinese Medicine (Approval No. IACUC-202306002). Under a normal light/dark (12 h/12 h) cycle, all rats were housed at a constant temperature of 25 ± 1 °C with a relative humidity of 60 ± 10%. In addition, all studies were conducted in strict accordance with the standards for laboratory animals established by the People’s Republic of China (GB 14922-2022) [48].

### 3.5. PCPA-Induced Insomnia Animal Model

SPF-grade SD male rats were randomly divided into 7 groups, 10 rats in each group, including the control group (CON, blank control), the model group (MOD), the diazepam group (DIA, positive control), the high-dose PC extract group (PC-H), the low-dose PC extract group (PC-L), the PC-80 fraction group (PC-80), and the N-acetyldopamine dimer A group (NADA). After 8 days of adaptive feeding, the rats in the control group were given 0.1 mL/(10 g/d) of distilled water by intraperitoneal injection and the other rats were intraperitoneally injected with 350 mg/(kg/d) of PCPA for two consecutive days. From the 11th day, rats in the control group and model group were administered 0.1 mL/(10 g/d) distilled water by gavage. Rats in the diazepam group were delivered 0.3 mg/mL of the diazepam solution. Rats in the PC-H group and the PC-L group were given 18.272 mg/mL and 4.568 mg/mL of the crude extract solution of PC, respectively. Rats in the PC-80 group were administered 4.576 mg/mL of the 80% methanol extract solution. Rats in the NADA group were administered 2.0154 mg/mL of the N-acetyldopamine dimer A solution. All rats were administered daily from 8 to 10 am for 7 consecutive days. The open-field behavioral experiment was performed at the end of the drug administration in each group, and the trajectory of the rats was recorded and analyzed by a rat behavioral analysis system (XinSoft Information Technology Co., Ltd., Shanghai, China).

### 3.6. HE Staining of Hypothalamic Sections

Rat hypothalamus tissues from each group were fixed in 4% paraformaldehyde, and these tissues were embedded and sectioned. Then, these sections were analyzed by HE staining. Microscopic examination of the images was carried out under an upright light microscope (NIKON ECLIPSE E100, Tokyo, Japan) produced by NIKON CORPORATION.

### 3.7. Enzyme-Linked Immunosorbent Assay (ELISA)

The hypothalamus tissues were processed to obtain homogenate, and then this homogenate was centrifuged at 4000 r/min for 10 min to obtain the supernatant. The content determinations of 5-HT, GABA, DA, BDNF, HVA, and DOPAC were carried out according to the instructions of the kits. Finally, the OD values were measured by microplate reader manufactured by TECAN (Infinite F50, Männedorf, Switzerland) with a 450 nm wavelength. According to the standard curve, the corresponding concentrations were determined.

### 3.8. Molecular Docking

The 2D structure of the active ingredient was downloaded from the pubchem database. The optimal 3D structure was converted using Chem3D with minimum free energy optimisation. Then, the small molecule was transformed into a pdbqt file by Auto Dock Tools. The 3D structure of the core target protein was downloaded from the PDB database (D2R PDB ID:6DW0). For protein receptors without mouse-derived crystal structures (GABA_A_, BDNF, and 5-HT1_A_), homologous modeling was performed using the online modeling tool Swiss Model. The protein receptors are subjected to hydrogenation, dehydration, and completion of missing residues using the ProteinPrep module in Maestro 13.0 software. The small molecule ligand is hydrogenated using the LigPrep module, saving as a sdf file, and then a docking grid is generated using the grid generation module. Finally, the molecular docking was performed using the Schrodinger’s Glide module SP algorithm to obtain the docking mode between small molecules and receptor proteins. A molecular dynamic simulation was conducted using the GROMACS 2024.1 package, adopting GROMACS 2024.1, with a GAFF force field for small molecules, a AMBER14SB force field for proteins, and an OPC water model for aqueous solvent modeling. After energy minimization and isothermal and isobaric pre-equilibrium treatment, the simulation system was finally simulated under the NPT ensemble for 100 ns, with a temperature of 298.15 K and a pressure of 1 bar.

### 3.9. Western Blot Analysis

Rat hypothalamic tissues with added lysis solution were homogenated to extract the protein. The homogenate solution was centrifuged to collect the supernatant containing the total amount of protein. The protein concentration was measured using the BCA protein concentration assay kit. Then, the total proteins were separated by SDS-poly-acrylamide gel (SDS-PAGE) and then transferred to polyvinylidene di-fluoride (PVDF) membranes. The PVDF membrane was closed with 5% milk on a shaker. After that, the PVDF membrane was incubated with the dilution of the primary antibody (ACTIN, DARPP-32, BDNF, and 5-HT1_A_) overnight at 4 °C. After being washed with TBST, the membranes were incubated with secondary antibodies (HRP goat anti-mouse, HRP goat anti-rabbit) for 30 min at room temperature. Finally, the PVDF membrane was completely immersed with the ECL reagents (Servicebio, Wuhan, China) for reacting for 1 min. The target protein blots were detected on chemiluminescence (CLINX, 6100, Shanghai, China). The grey scale values of the target proteins were analyzed and then compared with the internal reference as the relative expression of the target proteins. ACTIN protein was used as an internal reference.

### 3.10. Statistical Analysis

The results were expressed in the form of mean ± standard deviation ($\bar{X}$ ± S) and evaluated by IBM SPSS Statistics 26. One-way ANOVA was used to compare means between multiple groups and the LSD test was used as a post-hoc analysis; *p* < 0.05 was considered statistically significant.

## 4. Conclusions

Compared with plants and microorganisms, the research on animal drugs is still relatively weak, and the research on animal drugs mainly focuses on macromolecular proteins and peptides, which tends to neglect the potential role of small molecule compounds. The small molecule chemical compositions of 70% methanol PC extract contained acetyldopamine analogues, which were mainly enriched in the PC-80 fraction. The experiment proved that PC extract, the PC-80 fraction, and NADA could increase the levels of 5-HT, GABA, and BDNF, and reduce the levels of DA and its metabolites, HVA and DOPAC. Additionally, they also could affect the expression of 5-HT1_A_, BDNF, and DARPP-32 proteins. These neurotransmitters and related proteins were closely related to insomnia. Therefore, PC extract and NADA might ameliorate insomnia in rats by affecting 5-HT, GABA, and DA levels and 5-HT1_A_, BDNF, and DARPP-32 protein expression. The most effective fraction (PC-80 fraction) mainly consisted of small molecule compounds of N-acetyldopamine that were a kind of special component in insects. The results also have proved that acetyldopamine dimer A could significantly improve insomnia. It was speculated that the main pharmacological substances of PC extract in improving insomnia were acetyldopamine components. This study provided a basis for the high value utilization of PC, establishing a good theoretical basis for exploring functional drugs of the sleep-promoting function of PC.

## Figures and Tables

**Table 1:** Constituents identified from PC extract.

No.	t*_R_*	Molecular Formula	Molecular Ion	Measured Mass	Theoretical Mass	Error (ppm)	Fragment Ions	Type
1	4.18	C_20_H_22_N_2_O_6_	[M + H]^+^	387.1554	387.1556	−0.5	328.1155, 269.0792, 192.0642, 150.0539	dimers (I ^a^)
2	4.96	C_20_H_22_N_2_O_6_	[M + H]^+^	387.1537	387.1556	−4.9	328.1128, 269.0769, 192.0626, 150.0527	dimers
3	6.02	C_20_H_22_N_2_O_6_	[M + H]^+^	387.1587	387.1556	8	328.1187, 269.0819, 192.0662, 150.0555	dimers
4	6.67	C_20_H_20_N_2_O_6_	[M + H]^+^	385.1383	385.1394	−2.9	326.1012, 192.0651, 150.0547	dimers (II ^a^)
5	7.66	C_20_H_20_N_2_O_6_	[M + H]^+^	385.1404	385.1394	2.6	326.1029, 192.0663, 150.0556	dimers
6	7.90	C_30_H_31_N_3_O_9_	[M + H]^+^	578.2144	578.2139	0.9	150.0555	trimers
7	8.55	C_30_H_31_N_3_O_9_	[M + H]^+^	578.2180	578.2139	7.1	519.1774, 269.0813, 192.0661	trimers
8	8.82	C_30_H_31_N_3_O_9_	[M + H]^+^	578.2175	578.2139	6.2	192.0659	trimers
9	9.03	C_30_H_31_N_3_O_9_	[M + H]^+^	578.2201	578.2139	10.7	519.1771, 460.1391, 328.1181, 269.0810, 192.0658	trimers
10	9.27	C_30_H_31_N_3_O_9_	[M + H]^+^	578.2190	578.2139	8.8	519.1766, 460.1392, 387.1553, 328.1182	trimers
11	9.51	C_30_H_31_N_3_O_9_	[M + H]^+^	578.2190	578.2139	8.8	519.1768, 460.1388, 387.1554, 328.1185, 192.0658	trimers
12	9.88	C_30_H_31_N_3_O_9_	[M + H]^+^	578.2198	578.2139	10.2	519.1769, 460.1393, 328.1183, 269.0813, 192.0658	trimers
13	10.12	C_30_H_31_N_3_O_9_	[M + H]^+^	578.2156	578.2139	2.9	537.1866, 460.1377, 328.1179, 150.0554	trimers
14	10.39	C_30_H_29_N_3_O_9_	[M + H]^+^	576.1990	576.1982	1.4	537.1873, 385.1393, 192.0658, 150.0555	trimers
15	10.56	C_30_H_31_N_3_O_9_	[M + H]^+^	578.2171	578.2139	5.5	537.1865, 460.1385, 387.1549, 328.1181, 269.0812, 192.0658, 150.0553	trimers
16	10.87	C_30_H_29_N_3_O_9_	[M + H]^+^	576.1985	576.1982	0.5	537.1872, 385.1393, 192.0658, 150.0553	trimers
17	11.11	C_30_H_29_N_3_O_9_	[M + H]^+^	576.1989	576.1982	1.2	537.1867, 385.1391, 150.0553	trimers
18	11.38	C_40_H_40_N_4_O_12_	[M + H]^+^	769.2741	769.2721	2.6	576.1560, 385.1398, 326.1023, 150.0552	tetramers
19	11.52	C_40_H_40_N_4_O_12_	[M + H]^+^	769.2752	769.2721	4.0	576.1988, 385.1397, 326.1019, 150.0553	tetramers
20	11.79	C_40_H_40_N_4_O_12_	[M + H]^+^	769.2754	769.2721	4.2	576.1988, 385.1398, 150.0552	tetramers
21	11.96	C_40_H_40_N_4_O_12_	[M + H]^+^	769.2766	769.2721	5.9	571.1991, 385.1404, 192.0660	tetramers
22	12.00	C_40_H_40_N_4_O_12_	[M + H]^+^	769.2749	769.2721	3.6	576.1990, 385.1398, 326.1028, 192.0660	tetramers
23	12.30	C_40_H_40_N_4_O_12_	[M + H]^+^	769.2750	769.2721	3.8	576.1986, 385.1400, 326.1024, 150.0553	tetramers
24	12.54	C_40_H_40_N_4_O_12_	[M + H]^+^	769.2751	769.2721	3.9	576.1985, 385.1397, 192.0659	tetramers
25	12.68	C_40_H_40_N_4_O_12_	[M + H]^+^	769.2767	769.2721	6.0	385.1403, 150.0553	tetramers
26	13.09	C_40_H_40_N_4_O_12_	[M + H]^+^	769.2729	769.2721	1.0	576.1979	tetramers
27	13.19	C_40_H_40_N_4_O_12_	[M + H]^+^	769.2730	769.2721	1.2	576.1979, 192.0659	tetramers
28	13.87	C_50_H_49_N_5_O_15_	[M + H]^+^	960.3327	960.3303	2.5	767.2575, 593.1774	pentamers
29	14.25	C_50_H_49_N_5_O_15_	[M + H]^+^	960.3327	960.3303	2.5	767.2575, 578.2131	pentamers
30	14.80	C_50_H_49_N_5_O_15_	[M + H]^+^	960.3325	960.3303	2.2	767.2570, 576.1974, 387.1553	pentamers

^a^ Confirmed with reference compounds and structures of compounds I and II shown in Figure 1B.

**Table 2:** Effects of PC extract and NADA on brain index and kidney index in PCPA-induced insomnia rats.

Groups	Brain Index	Kidney Index
CON	0.60 ± 0.03	0.75 ± 0.06
MOD	0.63 ± 0.03 #	0.80 ± 0.03 #
DIA	0.66 ± 0.02 *	0.81 ± 0.06
PC-H	0.63 ± 0.03	0.81 ± 0.02
PC-L	0.65 ± 0.02	0.79 ± 0.05
PC-80	0.66 ± 0.01 *	0.81 ± 0.05
NADA	0.65 ± 0.02	0.88 ± 0.03 *

(Values are presented as the means ± standard deviation for each group, *n* ≥ 6 per group. Compared with the control group, #: *p* < 0.05; compared with the model group, *: *p* < 0.05.) Abbreviations: CON: control group; MOD: model group; DIA: diazepam; PC-H: high-dose PC extract group; PC-L: low-dose PC extract group; PC-80: PC-80 fraction group; NADA: N-acetyldopamine dimer A group.

## Data Availability

Data supporting reported results can be found from author.
